# Prostasin: An Epithelial Sodium Channel Regulator

**DOI:** 10.1155/2013/179864

**Published:** 2013-07-02

**Authors:** Shakti Aggarwal, Pradeep K. Dabla, Sarika Arora

**Affiliations:** ^1^Department of Biochemistry, ESI Hospital, Basai Darapur, New Delhi 110015, India; ^2^Department of Biochemistry, Chacha Nehru Bal Chikitsalaya, New Delhi 110031, India

## Abstract

Prostasin is a glycophosphatidylinositol-anchored protein which is found in prostate gland, kidney, bronchi, colon, liver, lung, pancreas, and salivary glands. It is a serine protease with trypsin-like substrate specificity which was first purified from seminal fluid in 1994. In the last decade, its diverse roles in various biological and physiological processes have been elucidated. Many studies done to date suggest that prostasin is one of several membrane peptidases regulating epithelial sodium channels in mammals. A comprehensive literature search was conducted from the websites of Pubmed Central, the US National Library of Medicine's digital archive of life sciences literature and the National Library of Medicine. The data was also assessed from journals and books that published relevant articles in this field. Understanding the mechanism by which prostasin and its inhibitors regulate sodium channels has provided a new insight into the treatment of hypertension and some other diseases like cystic fibrosis. Prostasin plays an important role in epidermal growth factor receptor (EGFR) signal modulation. Extracellular proteases have been implicated in tumor metastasis and local tissue invasion because of their ability to degrade extracellular matrices.

## 1. Introduction

Prostasin (also known as channel activating protease 1) is a novel extracellular serine protease with trypsin-like activity which cleaves synthetic substrates in vitro, preferentially at carboxy-terminal side of arginine residue. The serine proteases constitute one of the largest classes of proteolytic enzymes and have evolved to perform specialized functions. The catalytic triad that is essential for the enzymatic activity of prostasin is a histidine, aspartic acid, and serine sequence. Prostasin belongs to the classical serine protease family, with homology to trypsin, chymotrypsin, and kallikrein, and has a trypsin-like substrate specificity [1–3]. Trypsin-like serine proteases typically are synthesized as inactive zymogens that are activated by a single endoproteolytic cleavage. This group of enzymes often acts in either single or complex, highly regulated zymogen cascades to control important biological processes, such as coagulation, fibrinolysis, blood pressure, and digestion. The enzyme activation of prostasin occurs via cleavage of the proprotein to produce a light chain and a heavy chain that are disulfide linked [3]. Prostasin belongs to a distinct family of genes in syntenic regions of human chromosome 16p13.3/11.2 and mouse chromosomes 7 and 17 that includes tryptase-*γ* (*TPSG*), tryptase-*ε*, pancreasin, testisin, and distal intestinal serine protease [4]. In addition to being secreted, prostasin is expressed as a glycosylphosphatidylinositol- (GPI-) anchored membrane protein in prostate epithelial cells [5]. Soluble prostasin, purified from human seminal fluid, terminates at Arg323, suggesting that prostasin may be secreted via tryptic cleavage of its COOH terminal hydrophobic domain [1].

Prostasin was first identified as a product secreted by prostate gland and hence the name. Later on, techniques like immunolocalization, mRNA blotting, and in situ hybridization studies demonstrated that it was present in various other tissues like kidneys, lungs and airway, liver, pancreas, and salivary glands [2, 6, 7]. When characterization of cDNA allowed the prediction of the full precursor sequence, prostasin was recognized to be synthesized initially as a transmembrane protein with a C-terminal peptide anchor [2]. The C-terminal anchor is the defining characteristic of the type I transmembrane serine peptidases, a subset of vertebrate, trypsin-family peptidases [8, 9].

Prostasin is expressed mainly in mammalian epithelial tissues of prostate, kidney, lung, and colon as a 40 kDa glycosylphosphatidylinositol- (GPI-) anchored protein [1–3]. Although the biological and physiological roles of prostasin are not known at present, the high levels of prostasin in the prostate and seminal fluid (over 20-fold higher than any other tissue examined) suggest that it may play an important physiological role in these locations [10]. As evidenced from various studies, prostasin is present at low levels in other tissues, such as the lung, kidney, salivary glands, colon, liver, and bronchi, indicating that it may have roles in other biological processes as well [1]. In normal ovarian tissue, prostasin is not highly expressed [2, 11]. 

 A physiological function defined for prostasin is the proteolytic activation of the epithelial sodium channel (ENaC), an important regulator of sodium balance [7, 12, 13]. It is suggested that the full activation of ENaC is accomplished via a proteolytic cleavage of the ENaC gamma subunit by furin and a second cleavage presumably by prostasin [14]. Prostasin also plays important roles in maintaining the epidermal permeability barrier [15], regulating the inducible nitric oxide synthase gene expression during lipopolysaccharide-induced bladder inflammation [16], and regulating transepithelial resistance and current [4]. Prostasin has some additional features that are unusual in serine proteases, such as a high degree of sensitivity to monovalent and divalent cations, which may relate to the prostasin role in ENaC regulation [3].

Prostate cells can secrete and shed a fraction of prostasins as soluble enzymes with the rest remaining attached by a glycosylphosphatidylinositol (GPI) anchor [5]. However, the precise mechanism by which prostasin is secreted from cells has not been elucidated yet. Significantly, prostasin shares several similarities with a membrane-anchored frog protein, *channel-activating protease*, identified by expression cloning as a regulator of Na^+^ transport in *Xenopus *kidney cells [12, 13]. In a *Xenopus *oocyte expression of full length, but not COOH-terminally truncated, soluble prostasin/(CAP-1) results in activation of ENaC [13]. However, other investigators found that soluble, active prostasin can augment ENaC activity in M-1 kidney cortical collecting duct cells [17]. Additionally, in vitro invasion studies showed that overexpression of wild-type prostasin, but not a truncated secreted form of prostasin, inhibits cell invasion [18, 19]. These observations suggest that prostasin function may be regulated by membrane localization or secretion. However, the specific roles of the GPI anchor and COOH terminal domain have not been studied.

## 2. Structure of Prostasin

The crystal structure of the protease domain of prostasin is structurally similar to other serine proteases, consisting of two barrel-like subdomains with four conserved disulfide bonds. The two subdomains are separated by a cleft that contains the active site. In prostasin, the catalytic triad is formed by His-85, Asp-134, and Ser-238, corresponding to chymotrypsin residues His-57, Asp-102, and Ser-195. The proximity of the hydroxyl group of Ser-238 to the imidazole ring of His-85 and the hydrogen bond between O of Asp-134 and N of His-85 suggest that the catalytic mechanism observed in other serine proteases is conserved in prostasin [20].

In comparison with other serine proteases with known structures, the core of the domain is highly conserved, whereas the loops are less conserved. The sequence identity between the protease domain of prostasin and that of chymotrypsin, trypsin, elastase, and kallikrein is 37, 38, 32, and 39%, respectively [20]. Adjacent to the catalytic triad in serine proteases, there is a pocket that binds and confers specificity for the P1 position of substrates. In the structure of the prostasin apoprotein, this pocket is occluded by a loop containing residues 258–262 [20]. For chymotrypsin-like serine proteases, the specificity for the P1 position of the substrate is primarily determined by a residue at the bottom of the S1 pocket (analogous to Ser-189 in chymotrypsin). In prostasin, the equivalent residue is Asp-232, which is conserved in proteases characteristic of the trypsin family. This aspartate confers specificity toward lysine and arginine in the P1 position of substrates. In the structure of the 4-guanidinobenzoate ester adduct of prostasin, the terminal guanidinium group of the inhibitor forms hydrogen bonds with both carboxylate oxygens of Asp-232. Additional hydrogen bonds to the inhibitor are provided by the carbonyl oxygens of Ala-233, Asp-260, and Arg-267 [20].

 Shipway et al. [3] have extensively characterized prostasin substrate specificity. They have found that prostasin prefers an arginine or a lysine in the P1 position. The structure of the acyl-enzyme intermediate suggests that an arginine may be preferred over a lysine in the P1 position. The same group of researchers also found that metal ions regulate prostasin activity, and divalent cations show more potent inhibition. One of the proposed mechanisms for divalent cation-mediated regulation may be to affect the energetic favourability for the loop conformation in which the loop moves to block or expose the S1 pocket [20].

## 3. Inhibitors of Prostasin

The purified form of the human prostasin exhibiting serine protease activity can be inhibited by aprotinin, antipain, leupeptin, and benzamidine [1]. In the serpin-class serine protease inhibitors, protease nexin-1 (PN-1) [21, 22] also known as glia-derived nexin (GDN) or serpin E2 [23] is the cognate serpin for prostasin, forming a covalent complex with the latter to achieve inhibition of its serine protease activity. In the human PN-1 reactive site, the Leu-Ile-Ala-Arg residues at the P4-P1 positions of this suicide inhibitor are the optimal substrate for prostasin. A Kunitz-type reversible serine protease inhibitor, hepatocyte growth factor activator inhibitor 1 (HAI-1) is a physiologically relevant inhibitor of prostasin [24, 25], whereas HAI-2 can inhibit the prostasin protease domain in vitro [3]. The two isoforms of hepatocyte growth factor activator inhibitor 1 (HAI-1), probably originating from two mRNA splice variants of *HAI-1*, are *HAI-1A* and *HAI-1B* [24]. The synthetic serine protease inhibitor camostat mesilate and its active metabolite FOY-251 can inhibit prostasin serine protease activity in vitro [26] and possibly in vivo as well [27]. The enzymatic activity of prostasin is influenced by the levels of PN-1, HAI-1A, and HAI-1B and probably also by the expression levels of many yet undiscovered inhibitors. The relative physiological importance of PN-1, HAI-1A and HAI-1B as inhibitors of prostasin is at present unclear. It is possible that prostasin activity is regulated by the modulation of the ratio between HAI-1A and HAI-1B as it is not clear whether the two inhibitors have the same kinetic properties. HAI-1B differs from HAI-1A by a 16 amino acid insertion [28]. Prostasin is part of the matriptase-prostasin proteolytic cascade regulating terminal epidermal differentiation [29]. Matriptase is thought to be the first protease in the cascade due to its ability to autoactivate [30, 31] and because prostasin is activated by a matriptase-catalysed cleavage [29]. The downstream target for prostasin is unclear, but the matriptase-prostasin cascade eventually regulates the processing of the differentiation marker filaggrin [15, 32] and is essential for the establishment of epidermal integrity [15, 33]. 

At the time of its identification, the exact role of prostasin in mammalian physiology was not known, but various studies have been done all over the world which highlight its diverse roles in various biological and physiological processes. 

## 4. Role of Prostasin in Airway Clearance

Several lines of evidence suggest that one or more serine-class peptidases in vertebrate epithelia upregulate transcellular Na^+^ current mediated by ENaC, which is essential for airway fluid clearance [34]. For example, aprotinin, which is a broad-spectrum inhibitor of serine peptidases, reduces transepithelial Na^+^ transport in frog kidney cells [35, 36]. Aprotinin's target appears to be channel-activating protease. Similar studies in cultured mammalian airway cells show that amiloride-sensitive Na^+^ current is reversibly inhibited by aprotinin or bikunin (an inhibitor of tryptic serine peptidases) and restored by trypsin [5, 21, 34]. 

 The direct mechanism of protease-mediated ENaC activation is unclear, but features increased probability of channel opening [15, 21, 35]. Several membrane-associated serine peptidases in addition to prostasin are candidate physiological activators of ENaC in mammalian epithelia [15, 28, 37]. The physiological substrates of prostasin and the direct mechanism for ENaC activation by serine peptidases remain unknown. Patch clamp experiments suggest that prostasin increases open probability of ENaC, but there is no evidence that prostasin directly cleaves ENaC *α*-, *β*-, or *γ*-subunits [12]. Evidence implicating prostasin includes activation of ENaC by prostasin when mammalian versions of these proteins are coexpressed in frog oocytes [6, 28, 37]. Less direct evidence includes prostasin's sensitivity to inhibitors of ENaC-mediated Na^+^ current, like aprotinin [38], the finding that some epithelial cells that express ENaC also express prostasin [15], and phylogenetic evidence that prostasins are relatives of frog channel-activating protease [39].

**Figure 1:**
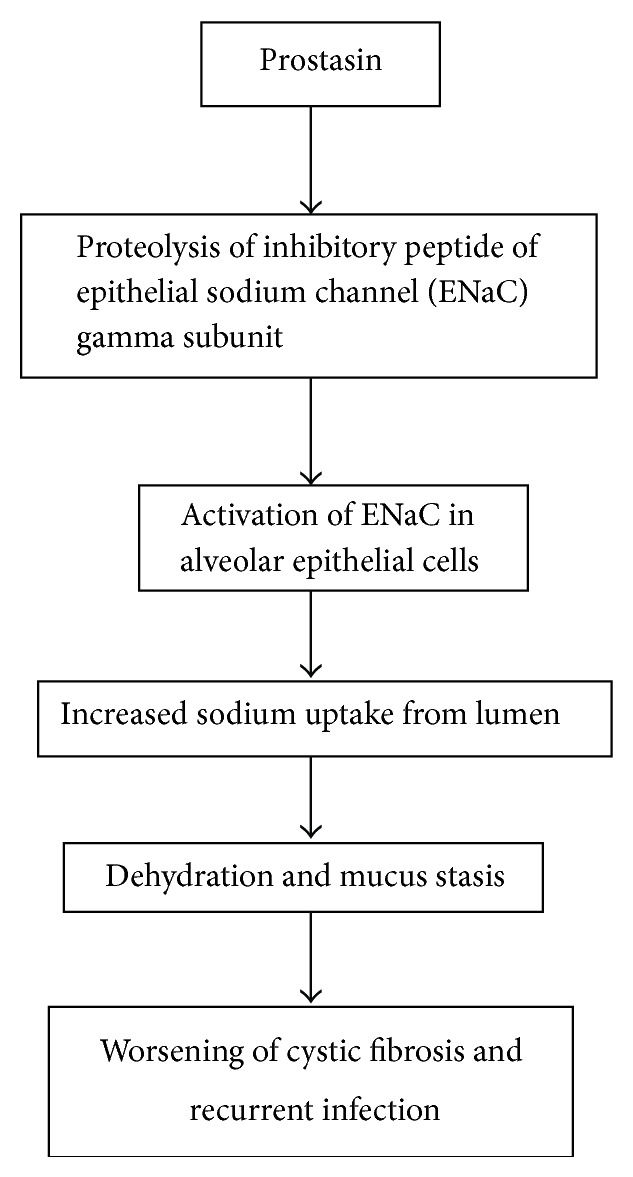
Role of prostasin in airway epithelium.

Electrophysiological studies of airway epithelia in cystic fibrosis (CF) suggest that Na^+^ uptake from the lumen is dysregulated and excessive [40–42]. The leading hypothesis regarding the cause of airways disease in CF is that excessive Na^+^ absorption leads to inadequate hydration resulting in mucus stasis and recurrent infection (Figure 1) and has prompted searches for ways to redress the postulated imbalance [14, 17]. Identification and inhibition of a proteolytic regulator of airway ENaC could provide a pharmacological means of improving Na^+^ current in CF airway. However, disordered regulation of ENaC by mutated CF transmembrane regulator protein may be the direct and dominant cause of the defect in CF [43].

In a study by Tong et al. in 2004, membrane-anchored prostasin was found to be highly expressed in CF epithelial cells, and the silencing of expression strongly reduced transepithelial Na^+^ current. This is direct evidence that prostasin is the major positive regulator of baseline ENaC activity in CF epithelium [44].

## 5. Role of Prostasin in Fluid and Electrolyte Regulation Actions at Renal Level

Epithelial sodium channel (ENaC) performs an essential function in several epithelial tissues, including the colon, kidney, and lung [7, 14, 45]. The epithelial sodium channel (ENaC) consisting of *α*-, *β*-, and *γ*-subunits is expressed in the renal distal tubules and constitutes the rate-limiting step for sodium reabsorption [46]. Two monogenic disorders, Liddle's syndrome (salt-retaining hypertension), and pseudohypoaldosteronism type 1 (salt-wasting hypotension) highlight the significance of genetic defects of ENaC in sodium homeostasis and blood pressure (BP) regulation [46]. Furthermore, genetic variants of *α*-ENaC, *β*-ENaC, and *γ*-ENaC were found to be associated with population BP variation or the presence of essential hypertension in some populations, although not in others [47–50].

In these tissues, sodium is primarily transported across the apical membrane via ENaC, and fluid transport across the membrane is highly responsive to sodium concentration. Several disease states like hypertension and cystic fibrosis can be affected by modulation of ENaC activity. Inappropriate function of ENaC leads to dysregulation of blood pressure in humans [51]. The same ENaC mutations which lead to hypertension in humans give rise to defects in lung fluid clearance in mice [51], suggesting that proper ENaC function may also be important in other diseases where sodium and fluid homeostasis is severely disrupted, for example, cystic fibrosis [52]. ENaC dysregulation leads to dehydration of airway surfaces in patients with cystic fibrosis, which in turn disrupts the primary innate lung defense mechanism, mucus clearance. In the last few years, a novel mechanism modulating ENaC activity by serine proteases was identified. Prostasin, one of the important serine peptidases, has been shown to regulate fluid and electrolyte regulation via proteolysis of the gamma (*γ*) subunit of epithelial sodium channel [7, 14]. Cleavage of an inhibitory peptide in the *γ*-ENaC subunit by prostasin has been shown to stimulate sodium transport by 2-3-fold in cell-based experiments [7, 14]. Degree of proteolytic activation of ENaC is modulated by the balance between channel-activating protease activity and soluble protease inhibitor concentrations present on the apical surface of airway epithelium [34, 53]. A study done by Myerburg et al. [43] demonstrated that abnormal prostasin regulation in CF epithelia leads to excessive proteolytic activation of ENaC and that this plays a significant role in the Na^+^ hyperabsorption characteristic of CF airway disease. Further defining of the regulation of proteolytic cascades on the airway cell surface may reveal novel targets for CF therapeutics beyond the protease inhibitors currently under trial. 

**Figure 2:**
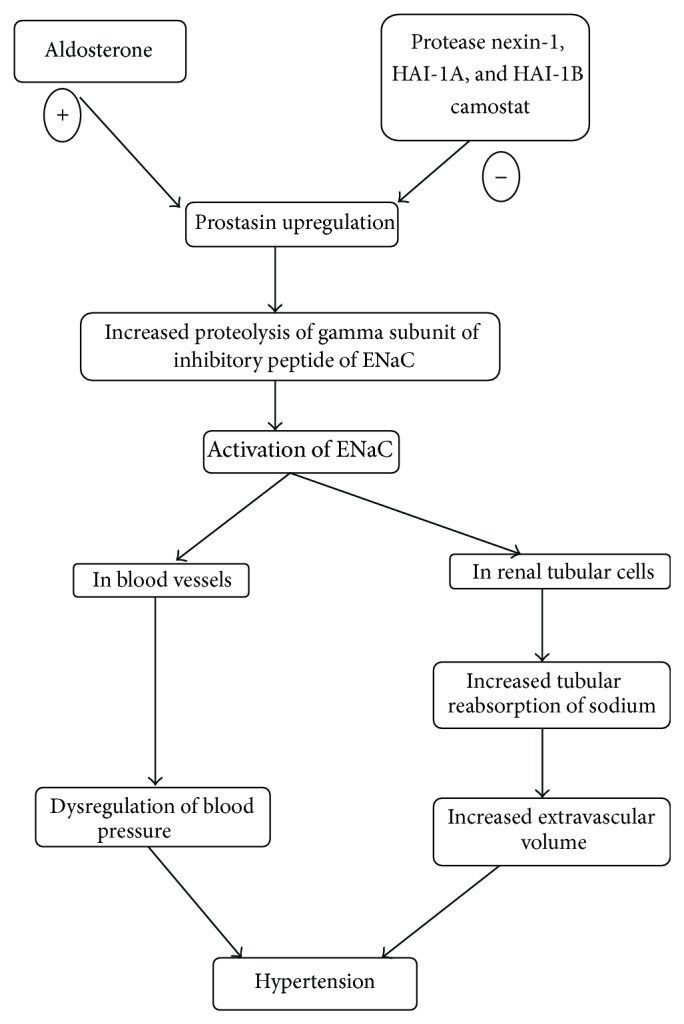
Role of prostasin in hypertension.

Another possible role of prostasin in maintenance of fluid and electrolytes is aldosterone dependant. Prostasin seems to be involved in aldosterone-dependent ENaC activation at renal level; however, it is unclear whether aldosterone increases prostasin expression or, on the contrary, a preceding stimulation of prostasin determines adrenal secretion of aldosterone (Figure 2). In 2002, Narikiyo et al. [17] demonstrated that aldosterone substantially increases prostasin expression, with functional consequences on Na^+^ balance in vivo; moreover, they reported that urinary excretion of prostasin in 3 patients with aldosterone-producing adenoma was abnormally elevated and normalized after adrenalectomy. These findings suggested urinary prostasin as a possible marker of aldosterone-dependent ENaC activation at the renal level in humans. However, in 2003, Wang et al. showed that expression of human prostasin by adenovirus-mediated gene delivery was associated with subsequent increase of aldosterone and, in turn, of blood pressure levels in rats [35]. Elevated plasma aldosterone levels were detected at 3 days after gene transfer before the development of hypertension, indicating that adrenal stimulation of mineralocorticoid production by prostasin is the primary event and not vice versa [35]. Prostasin regulates aldosterone production in human adrenocortical H295R cells by a protease-independent but calcium- and protein-kinase-C- (PKC-) dependent mechanism [54].

Olivieri et al. evaluated the physiological relationship linking prostasin, aldosterone, and ENaC function and proposed the role of urinary prostasin as a candidate marker for epithelial sodium channel activation in humans [55]. For this study, young students without any significant history of disease and in apparently good health, with proven normal blood pressure and normal plasma potassium, free of any hormone- or drug-related interference, were studied before and after spironolactone. A single dose of spironolactone (100 mg) was given to induce an effective competitive inhibition of aldosterone action at mineralocorticoid receptor level and, in turn, of subsequent Na^+^ transport through the ENaC. Single dose of spironolactone increased urinary Na^+^/K^+^ ratio and decreased urinary prostasin expression. However, these effects were not generalized but were restricted to the group of individuals in whom the renin/aldosterone axis was activated by a relatively low sodium intake. Thus, in agreement with the expected inhibition of aldosterone and ENaC function, spironolactone-dependent changes in urinary Na^+^ and K^+^ excretion were proportional to the hormone levels, resulting as relevant or null in subjects with high or low aldosterone, respectively. Similarly, a spironolactone-inhibited aliquot of prostasin was observed only in individuals with an activated renin/aldosterone axis.

Koda et al. developed a specific radioimmunoassay (RIA) for human prostasin to clarify the physiological relationship among prostasin, aldosterone, and ENaC. Prostasin levels in urine were determined in 26 normotensive and 121 hypertensive subjects. Aldosterone content in urine and plasma, urinary Na/K ratio, and other clinical parameters were also measured. A highly significant correlation between prostasin and aldosterone concentrations in urine (correlation coefficient: 0.673, *P* < 0.0001) was observed in this study. A significant correlation was also found between urinary prostasin and plasma aldosterone concentrations (correlation coefficient: 0.229, *P* < 0.05). In addition, urinary prostasin excretion was inversely correlated with urinary Na/K ratio (correlation coefficient: −0.425, *P* < 0.0001). These findings suggest that urinary prostasin level is strongly correlated with urinary or plasma aldosterone level and may serve as a surrogate marker for ENaC activation in hypertensive patients [56]. 

The increase in the levels or function of prostasin caused by functional genetic variants might amplify or result in the prostasin-dependent release of an inhibitory peptide from *γ*-ENaC, subsequently cleaving the channel and causing an increased channel gating or open probability [14]. ENaC activation in the distal nephron augments tubular sodium reabsorption and extracellular volume expansion, which leads to BP elevation.

## 6. Role of Prostasin in Epithelial Cancers

**Figure 3:**
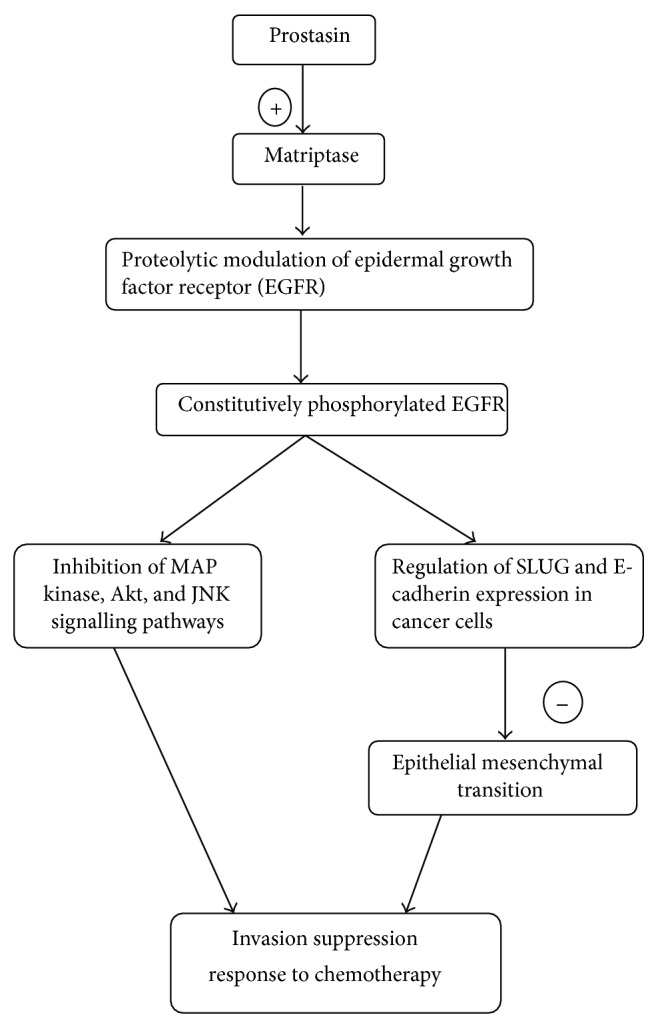
Role of prostasin in cancers.

Prostasin's role in cancer is unclear, but there is evidence indicating that it may be related to tumor promoter mechanisms [26] as well as tumor suppressor mechanisms [18, 19, 38]. Recently, other data has also shown that prostasin is involved in proteolytic cleavage of the extracellular domain of epithelial growth factor receptor (EGFR), causing a constitutively phosphorylated receptor [41] that could potentially participate in promoting tumor growth (Figure 3).

## 7. Epidermal Growth Factor Receptor (EGFR) Signal Modulation

The epidermal growth factor receptor (EGFR) is the cell surface receptor for members of the epidermal growth factor family (EGF family) of extracellular protein ligands [36]. It exists on the cell surface and is activated by the binding of its specific ligands. Upon activation by its growth factor ligands, EGFR undergoes a transition from an inactive monomeric form to an active homodimer—although there is some evidence that preformed inactive dimers may also exist before ligand binding. EGFR dimerization stimulates its intrinsic intracellular protein-tyrosine kinase activity. As a result, autophosphorylation of several tyrosine (Y) residues in the C-terminal domain of EGFR occurs. This autophosphorylation elicits downstream activation and signaling by several other proteins that associate with the phosphorylated tyrosines through their own phosphotyrosine-binding SH2 domains. These downstream signaling proteins initiate several signal transduction cascades, principally the mitogen activated protein kinase (MAPK), Akt, and JNK pathways, leading to DNA synthesis and cell proliferation [39]. Such proteins modulate phenotypes such as cell migration, adhesion, and proliferation. Mutations, amplifications, or misregulations of EGFR or family members are implicated in various epithelial cancers. Mutations involving EGFR could lead to its constant activation which could result in uncontrolled cell division—a predisposition for cancer.

**Figure 4:**
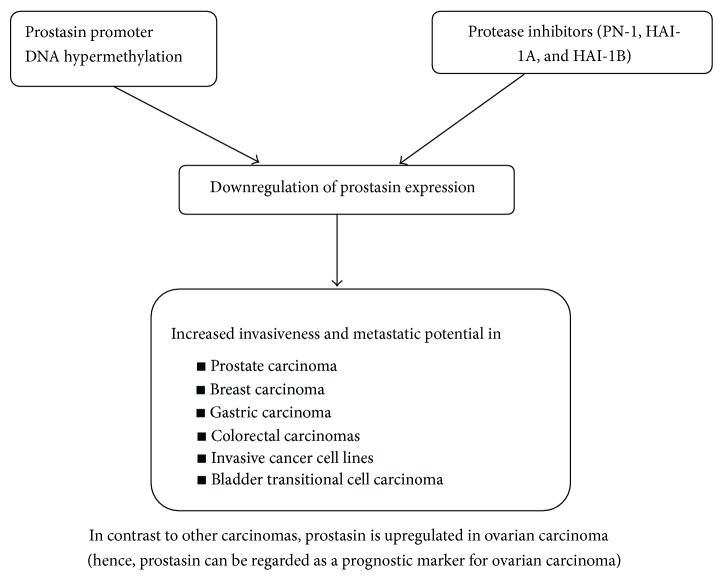
Regulation of prostasin expression in carcinomas.

Extracellular proteases and protease inhibitors are believed to play an important role during carcinogenesis in many different ways, such as degrading the extracellular matrix in order to facilitate invasive growth and activating signal molecules. In accordance with this, proteases were mostly thought of as promoters of metastasis. However, clinical trials where cancer patients were treated with broad range protease inhibitors have shown that proteases can act as tumor suppressors [40]. Prostasin, a relatively new protease, is found to be involved in the extracellular proteolytic modulation of the epidermal growth factor receptor (EGFR) and is thus an invasion suppressor. Prostasin, which is found to be expressed abundantly in normal epithelia and essential for terminal epithelial differentiation [15], has been seen to be downregulated in human prostate, breast, and gastric cancers and invasive cancer cell lines (Figures 3 and 4). Fu et al. [37] investigated the impact of prostasin expression regulation on this cellular function as well as the molecular mechanisms involved in human cytotrophoblastic cells. The results of this study showed that prostasin may regulate trophoblast cell proliferation via modulating the EGFR-MAPK signaling pathway.

Recent advances in bladder cancer research have identified the process of epithelial-mesenchymal transition (EMT) as an important factor in determining disease prognosis and patient responses to therapy. EMT is known to be the main cause in the development of invasive and metastatic cancers including transitional cell carcinoma (TCC). At the molecular level, the epidermal growth factor receptor (EGFR), the cell adhesion molecule E-cadherin, and transcription repressors of E-cadherin, such as SNAIL and SLUG, have been shown to play essential and major roles in EMT and development of invasive and metastatic bladder cancer [57]. A lot of work has been carried out by Chen et al. who demonstrated that a glycosylphosphatidylinositol- (GPI-) anchored epithelial extracellular membrane serine protease, prostasin/PRSS8, modulates EGFR signalling via enhancement of matriptase cleavage of the EGFR extracellular domain (ECD) and regulates SLUG and E-cadherin expression in cancer cells [41, 42]. In their other study, the authors have showed [58] that loss of prostasin expression in bladder transitional cell carcinomas is associated with epithelial-mesenchymal transition (EMT) and may have functional implications in tumor invasion and resistance to chemotherapy [58]. Downregulation of prostasin protein expression has also been shown to be associated with high-grade and hormone-refractory prostate cancers [19, 38], breast cancers [59], and gastric cancers [60]. Prostasin promoter DNA hypermethylation has been shown to be an epigenetic mechanism of prostasin silencing in various cancer cell lines [18, 22, 60]. Invasive human cancer cell lines are often associated with loss of prostasin expression, while prostasin reexpression inhibits their invasion through Matrigel [18, 19].

In a study by Takahashi et al., expression levels of both prostasin mRNA and protein were found to be inversely correlated with histological differentiation but not associated with clinical stage of human prostate cancer. Almost all cases of metastatic and hormone-refractory cancers demonstrated down-regulation of prostasin expression, indicating that prostasin may not be regarded as a prognostic marker for prostate cancer but a useful marker for tumor differentiation [38]. Chen et al. in 2001 showed that both prostasin protein and mRNA were expressed in normal human prostate epithelial cells and noninvasive human prostate cancer cell line, the LNCaP, but neither was found in invasive human prostate cancer cell lines DU-145 and PC-3. Prostasin mRNA expression was absent in invasive prostate cancer cell lines of a transgenic mouse model. Immunohistochemistry analysis also showed that prostasin protein expression is down-regulated in high-grade prostate cancer [19].

In experimental studies on breast cancer cell lines by Chen and Chai [18], it has been shown that prostasin mRNA and protein were expressed in normal human mammary epithelial cells (NHMEC), the poorly invasive breast carcinoma cell line MCF-7, and the nonmetastatic breast carcinoma cell line MDA-MB-453, but absent in highly invasive and metastatic breast carcinoma cell lines MDA-MB-231 and MDA-MB-435s. Enforced reexpression of prostasin in MDA-MB-231 and MDA-MB-435s reduced the in vitro invasiveness of either cell line by 50%. Examination of the prostasin gene promoter and first exon revealed a GC-enriched region that contains transcription regulatory elements. The promoter and exon 1 region of the prostasin gene was investigated for DNA methylation in NHMEC and the carcinoma cell lines. The results revealed a methylation pattern that correlates with prostasin expression in these cells. Demethylation coupled with histone deacetylase inhibition resulted in reactivated expression of the prostasin mRNA in MDA-MB-231 and MDA-MB-435s cells. These results suggest that prostasin expression in breast cancer cells may be regulated by DNA methylation and that an absence of prostasin expression may contribute to breast cancer invasiveness and metastatic potential. In another study by Bergum et al. in 2012, a coordinated coexpression pattern has been reported for prostasin and its activating enzyme/substrate matriptase in human breast cancers [61]. Furthermore, matriptase and prostasin displayed a near identical spatial expression pattern in the epithelial compartment of breast cancer tissue [61].

Selzer-Plon et al. investigated the mRNA levels of prostasin and its inhibitors PN-1, HAI-1A, and HAI-1B, during colorectal cancer carcinogenesis in humans. Their data demonstrated that elevated mRNA levels for prostasin inhibitor, PN-1, coincides with the acquisition of malignant properties, whereas the mRNA level of prostasin is relatively stable during colorectal cancer carcinogenesis [62]. It shows that the action of proteases may be regulated not necessarily by the expression of the protease but by regulating the expression of its inhibitors. It could thus be speculated that the transition from severe dysplasia to cancer is accompanied by a transcriptional decrease in prostasin expression and/or a downregulation of prostasin activity via upregulation of one or more enzymatic inhibitors of prostasin. In a study by Cavalieri et al., prostasin mRNA expression was found to be correlated to prolonged survival after surgery in colon cancer patients [63].

Unlike bladder and colon cancer, an upregulation of prostasin has been reported in ovarian carcinoma [64, 65]. The potential use of prostasin as a marker of ovarian cancer has been suggested in a study by Mok et al. [64] that demonstrated 120- to 410-fold higher prostasin mRNA expression in ovarian cancer cell lines than normal ovarian cell lines. The abnormal expression of prostasin in these cell lines was further confirmed at the protein level by immunostaining. They found higher levels of serum prostasin in case patients with ovarian cancer than in control subjects and declining levels of prostasin after surgery for ovarian cancer [64]. The poor prognosis associated with ovarian cancer is mainly due to the fact that the majority of patients are diagnosed in the advanced stages of the disease.

## 8. Conclusion

Prostasin, a serine protease capable of regulating epithelial sodium channel, seems to have a vast potential as a biomarker in cystic fibrosis and hypertension. Further studies and research in this area will highlight the potential role of prostasin as a screening marker for cancers or a therapeutic agent for treating invasive and chemoresistant tumors.
